# Productive Performance and Some Biochemical Indices of Ossimi Ewes and Their Lambs to Dietary Inclusion of Selenium, Zinc Nanoparticles, or Their Combination

**DOI:** 10.3390/ani15182694

**Published:** 2025-09-15

**Authors:** Emadeldien Mohamed Ibrahim, Yasser Alrauji, Shaaban S. Elnesr, Mohamed Shehab-El-Deen

**Affiliations:** 1 Animal and Poultry Production Department, Faculty of Agriculture, Minia University, El-Minya 61519, Egypt; 2 Department of Animal and Poultry Production, College of Agriculture and Food, Qassim University, Buraydah 51452, Al-Qassim, Saudi Arabia; m.shehabeldeen@qu.edu.sa; 3 Department of Poultry Production, Faculty of Agriculture, Fayoum University, Fayoum 63514, Egypt; ssn00@fayoum.edu.eg; 4 Department of Animal Production, Faculty of Agriculture, Suez Canal University, Ismailia 41522, Egypt

**Keywords:** nanominerals, digestibility, performance, metabolites, antioxidants, sheep

## Abstract

This study aimed to evaluate the effects of dietary nano-selenium (Se-NP), nano-zinc (Zn-NP), and their combination, on the performance of Ossimi ewes and their offspring. Twenty-eight pregnant Ossimi ewes were randomly allotted to one of four equal experimental groups. The ewes were fed a basal diet with the addition of 0.3 mg selenium nanoparticles (Se-NP), 30 mg zinc (Zn-NP), or 0.3 mg Se-NP plus 30 mg Zn-NP (SZ-NP)/kg DM. The results showed that nutrient digestibility, nutritive values, milk yield, and fat corrected milk, as well as milk constituents yields, were improved (*p* < 0.05) for ewes fed Se-NP, Zn-NP, or SZ-NP vs. the control one. As well, lambs’ birth weight, final body weight, and average daily gain were increased (*p* < 0.05). Serum immunoglobulin G, total protein, albumin, globulin, and glucose values were higher (*p* < 0.05); however, serum cholesterol level tended to be decreased. Ewes and their respective lambs in the Se-NP, Zn-NP, or SZ-NP groups had lower (*p* < 0.05) urea concentrations and liver enzyme activity than the control. Thyroid hormones, total antioxidant capacity, and glutathione peroxidase activity were higher (*p* < 0.05) in the Se-NP-, Zn-NP-, or SZ-NP-fed groups. This improvement was accompanied by favored growth performance, immune function, and overall health in their suckling lambs, with selenium being more effective than zinc. In conclusion, the dietary inclusion of selenium, zinc nanoparticles, or their combination can be an effective strategy to enhance productivity and health in ewes and their offspring.

## Simple Summary

Feed additives can boost ruminant performance by optimizing rumen function and nutrient absorption. Nowadays, nanotechnology offers advanced solutions for nutrient delivery and a large potential to improve animal production systems. This potential is primarily attributed to the unique physicochemical properties of nanoparticles, including their small size (1–100 nm), stability, high absorption efficiency, and large surface area. Nanominerals, such as selenium and zinc, improve bioavailability, reduce waste, and enhance digestibility, productivity, growth, immunity, and antioxidant activity. The present study was conducted to evaluate the effects of selenium, zinc nanoparticles, or their combinations, upon nutrient digestibility, productive performance, some biochemical indices, and antioxidant status of Ossimi ewes and their suckling lambs. The results indicate that the inclusion of selenium nanoparticles, either alone or in combination with zinc nanoparticles, exerts a more pronounced effect on ewe nutrient utilization, productivity, and antioxidant levels, which leads to better growth performance, immune function, and overall health in their suckling lambs than zinc nanoparticles alone.

## 1. Introduction

A well-balanced ration containing adequate energy, protein, minerals, and vitamins is essential for animal growth, production, and reproduction. Trace minerals, despite their being required in minute quantities, play vital roles in numerous physiological processes, such as the maintenance of animal growth, and productive and reproductive performance, as well as health status [1]. Ensuring adequate trace mineral supplementation is crucial to prevent significant economic losses in livestock production. Selenium (Se) and zinc (Zn) are essential trace minerals directly influencing growth performance, nutrient utilization, reproductive efficiency, and immune function in ruminants [2,3].

Selenium plays vital roles in numerous biochemical pathways critical for animal health and metabolism [4]. For instance, Se acts as a catalytic center for glutathione peroxidase [5], and serves as a redox center in thioredoxin reductase [6]. It is an essential cofactor for iodothyronine deiodinases that regulate thyroid hormone activation [7,8]. So, selenium concentration must be adequate in animal feed. Hence, Se deficiency symptoms are widespread in ruminants [9,10]. Metabolic disorders caused by Se deficiency led to lower productivity, health disorders, and even the death of animals, and, consequently, producer losses [11]. As well, Se deficiency in ewes causes muscular dystrophy in their lambs and contributes to ewe fertility issues, abortions, and retained placenta. Moreover, during the third trimester, accelerated fetal growth increases maternal selenium transfer to the fetus. This means that insufficient Se intake in pregnant/lactating ewes can induce deficiency in both ewes and their newborns, impairing immunity and productivity [12]. Furthermore, newborn lambs from deficient mothers face higher mortality, reduced vitality, impaired suckling, and weakened immunity, increasing infection risk [12,13].

Zinc (Zn) is crucial for ruminants, playing catalytic, structural, and regulatory roles in enzymes, proteins, and transcription factors [14]. It is involved in metabolism, growth, immunity, and antioxidant defense systems [15,16], with approximately 300 enzymes that are related to metabolism, carbohydrate production, and other biochemical processes [17]. Accordingly, Zn supplementation is crucial for boosting immunological functions and preventing viral and metabolic illnesses [18], as well as improving nutrient digestibility [19]. In addition, some studies demonstrated that Zn supplementation enhances milk production and blood immunoglobulin levels in ruminants [20,21].

Traditionally, these minerals are supplemented in animal diets in inorganic forms (e.g., sodium selenite and zinc oxide) or organic forms (e.g., selenium-yeast and zinc proteinate). While an improvement over inorganic sources, organic minerals can be costly. A significant limitation common to both traditional forms is their suboptimal bioavailability; inorganic minerals are particularly susceptible to antagonisms in the digestive tract, resulting in low absorption rates, higher required doses, and a potential for environmental pollution. This has driven the search for more advanced and effective forms of mineral delivery [4,8,10,15].

Nanomineral particles, particularly Se and Zn with diameters of 1–100 nm, offer significant advantages in animal nutrition due to their unique physicochemical properties such as small size, high stability, hydrophobicity, and large surface area-to-volume ratio [22,23]. Such properties enable efficient dispersion in biological media, enhanced cellular uptake, greater mucosal tissue interaction, prolonged gastrointestinal residence time, and faster diffusion into cells compared to larger particles [22,24]. The higher bioavailability of nanominerals improves nutrient digestibility, antioxidant capacity, growth, immunity, reproduction, and overall health in ruminants [23,25,26,27]. Thereby, Se and Zn nanoparticles are recognized as a sustainable, low-toxicity alternative to traditional sources of Se and Zn, demonstrating superior efficacy to enhance those traits [28,29]. Thus, Se and Zn nanoparticles provide innovative solutions for targeted nutrient delivery and health protection, and, consequently, enhanced animal productivity. In sheep, comparative invivo data for nano-Se vs. nano-Zn are not well documented and need to be evaluated. So, this study was designed to clarify how the dietary inclusion of Se, Zn nanoparticles, or their combination could affect ewes’ nutrient utilization, milk production, metabolic profile, and antioxidant status, as well as the performance of their offspring.

## 2. Materials and Methods

All animal procedures were approved by the Ethical Committee for the Care and Use of Animals in Education and Scientific Research, Faculty of Agriculture, Minia University, El-Minya, Egypt, under approval No. MU FA 0321123.

### 2.1. Animals Feeding and Management

Twenty-eight Ossimi ewes at late gestation (4–6 weeks prepartum), averaging 49.68 ± 1.85 kg, were selected for this study because this period is characterized by accelerated fetal growth and significantly increased maternal-to-fetal transfer of nutrients, making it a critical window where mineral supplementation is most impactful for the health of both ewes and their offspring. The study was conducted at the experimental farm of the Animal Production Department, Faculty of Agriculture, Minia University, located in El-Minia, Egypt. Ewes received a concentrate feed mixture (CFM) to meet their nutrient requirements based on their live body weight [30]. The ewes were randomly allocated into four equal groups of similar initial body weights in a completely randomized design. They were fed on a basal diet (control) with the addition of 0.3 mg Se nanoparticles (Se-NP), 30 mg Zn nanoparticles (Zn-NP), or 0.3 mg Se-NP plus 30 mg Zn-Np (SZ-NP)/kg DM. The requirements of Se and Zn are 0.3 mg/kg diet and 30 mg/kg diet, respectively, [30]. The Se-NP and Zn-NP used in the study were obtained from Sigma-Aldrich, St. Louis, MO, USA. Both were of feed grade, in powder form, and had a purity of 99.9%. The Se-NP had a particle size of less than 80 nm, while the Zn-NP particles ranged between 60 and 80 nm. These nanoparticles were first thoroughly premixed with finely ground yellow corn before being incorporated into the other components of the CFM. The CFM consisted of wheat bran (40), yellow corn (30), soybean meal (17), wheat straw (10), calcium carbonate (2), and sodium chloride (1). Alfalfa hay (AH) was provided as the roughage component offered ad libitum throughout the experimental period.

The ewes were maintained in enclosed stable facilities throughout the feeding trial. Dietary supplementation began during late gestation (4–6 weeks prepartum) and continued throughout the three-month lactation period. Animals receive their allocated feed twice daily (08:00 and 14:00 h) with continuous access to fresh drinking water.

### 2.2. Dietary Sampling and Laboratory Analysis

Dry matter intake (DMI, kg/head/day) was measured weekly during the final week of each month and considered in the calculation of nutrient digestibility and feeding values. Ewe body weights (kg) were recorded during late gestation, at lambing and throughout the 3-month lactation period. Lamb birth weights (kg) were measured within 24 h postpartum and biweekly during the 3-month suckling phase. Lambs’ average daily gain (ADG, g/day) was calculated from growth data. Daily feed samples were collected during the final week of each experimental month, and a composite sample was prepared for analysis, and was split into two portions: the first one dried at 105 °C to constant weight for DM determination; the second was dried at 70 °C, ground, and stored in airtight containers for chemical analysis. Dietary samples were analyzed for dry matter (DM), organic matter (OM), crude protein (CP), crude fiber (CF), ether extract (EE), and ash [31]. Fiber fractions (neutral detergent fiber, NDF and acid detergent fiber, ADF) were determined [32]. Fecal samples were collected twice daily (07:00 and 14:00) during the final week of each month, dried at 70 °C to constant weight, and analyzed for DM, OM, CP, CF, NDF, ADF, EE, and ash. Total tract digestibility (%) was determined using acid-insoluble ash as an internal marker [33]. Chemical composition of experimental rations is presented in Table 1:

### 2.3. Milk Sampling and Analysis

Daily milk yield (g/head/d) was measured biweekly from each ewe starting from day 5 post-lambing until weaning. Daily milk yield was determined by using the lamb sucking technique [34]. Lambs were separated from their mothers at 4.0 p.m. on the day before measuring milk yield. The following day, lambs were weighed at 8.0 a.m., and left to suckle their dams till satisfaction, then reweighed and kept away from their mothers. The residual milk in the udder of each dam was hand milked and weighed. At 4.0 p.m., the lambs were weighed again before and after suckling, and the residual milk in the udder was also hand milked and weighed. The amount of milk consumed by each lamb in the morning and afternoon was calculated by the differences between the weights recorded before and after suckling. The ewes’ hand-milked yield (in the morning and afternoon) was added to the daily milk intake by her suckling lambs to give an estimate of 24 h milk production. Biweekly, representative milk samples were individually collected post-lambing throughout lactation. Milk samples were analyzed via infrared spectrophotometry (MilkoScan 130 series, FOSS Electric, Hillerød, Denmark) for percentages (%) and yields (g/d) of protein, fat, lactose, and solid-not-fat (SNF) content. Fat-corrected milk yield (FCM, g/head/day) was calculated [35].

### 2.4. Blood Sampling and Biochemical Analysis

Non-heparinized blood samples were obtained from the jugular vein of all ewes and their offspring at 12, 24, and 48 h, and biweekly, thereafter before access to feed or drink. Samples were allowed to clot at room temperature (≥4 h), followed by centrifugation at 1500× *g* for 20 min to separate serum. Cleared sera were stored at −20 °C until analysis. Serum total protein (g/dL), albumin (g/dL), glucose (mg/dL), cholesterol (mg/dL), aspartate transaminase (AST, U/L), Alanine aminotransferase (ALT, U/L), and urea (mg/dL) were determined calorimetrically (Bio-diagnostic kits, Biodiagnostics, Cairo, Egypt). Serum globulin concentrations were calculated (Globulin (g/dL) = Total protein − Albumin). Levels of immunoglobulin G (IgG, mg/mL) were quantified via bovine-specific ELISA kits (Alpha Diagnostic International, San Antonio, TX, USA, and Kamiya Biomedical, Seattle, Washington, DC, USA) at 12, 24, and 48 h post-lambing. Serum triiodothyronine (T_3_, nmol/L) and thyroxine (T_4_, nmol/L) concentrations were measured using ELISA (BioCheck EIA kits, Foster City, CA, USA); the T_3_/T_4_ ratio was then calculated. Serum total antioxidant capacity (TAC, mM/L) and glutathione peroxidase (GSH-Px, U/mL) activities were analyzed using STAT-LAB SZSL60-SPECTRUM (Bio-diagnostic kits, Dokki, Giza, Egypt).

### 2.5. Statistical Analysis

A randomized block experimental design with repeated measurements were subjected to one way ANOVA using the general linear model (GLM) procedure of SAS, version 9.1 [36] with a model Yij = µ + Ti + eij, where Yij is the parameter under analysis, and µ is the overall mean; Ti is the effect of ith treatments and eij is the experimental error. Tukey’s procedure for multiple comparisons was used to compare the differences between the means. Statistical differences among means were considered significant at *p* < 0.05. Data are presented as means ± pooled standard error of the mean (SEM), with exact *p*-values reported where applicable.

## 3. Results

### 3.1. Digestibility and Feeding Values

The data in Table 2 illustrate the dietary inclusion effects of selenium nanoparticles (Se-NP), Zinc NP (Zn-NP), and selenium–zinc nanoparticles (SZ-NP), either separately or in combination, upon nutrient digestibility and nutritive values. The results reported that the DM, OM, CP, EE, CF, NDF, and ADF digestibility values as well as the nutritive values were increased (*p* < 0.05) for ewes fed Se-NP, Zn-NP, or SZ-NP when compared to the control. Also, nutrient digestibility and nutritive values significantly improved for ewes that received Se-NP and SZ-NP diets compared to those received Zn-NP alone.

### 3.2. Productive Performance

In ewes, data in Table 3 indicates that the differences in IBW, FBW, and DMI were not significantly altered among treatments. Meanwhile, DCPI and TDNI increased (*p* < 0.05) in ewes that received Se-NP, Zn-NP, or SZ-NP compared to the control. In the case of the lambs, there was an increase (*p* < 0.05) in BW by 6.64%, 19.22%, and 43.24% for lambs born to ewes that received Se-NP, Zn-NP, or SZ-NP vs. control, respectively. Additionally, BW was significantly improved by 14.61% and 20.15% for lambs born to ewes that received Se-NP or SZ-NP compared to those that received Zn-NP. Data showed higher (*p* < 0.05) increases in FBW and ADG for lambs born to ewes that received Se-NP (32.56% and 19.78%), Zn-NP (35.17% and 31.81%), or SZ-NP (21.30% and 33.52%) than the respective control. In addition, there was also an improvement (*p* < 0.05) in FBW and ADG, amounting to 10.67 and 12.85, and 8.67 and 10.07 for lambs born to ewes that received Se-NP or SZ-NP, respectively, vs. Zn-NP alone.

### 3.3. Milk Yield and Composition

As shown in Table 4, there were increases (*p* < 0.05) in MY and FCM, amounting to 47.55, 32.50, and 49.17, and 49.71, 33.47, and 51.90% for ewes that received Se-NP, Zn-NP, or SZ-NP, respectively, vs. the control. Milk yield and FCM values were greater (*p* < 0.05) by 11.36, 12.59 and 12.17, 13.81%, respectively, for ewes that received Se-NP or SZ-NP vs. Zn-NP alone. Feed efficiency (FCM/DMI) showed more improvement (*p* < 0.05) in ewes that received Se-NP, Zn-NP, or SZ-NP compared to the control. As well, milk fat, protein, and lactose yields (g/d) have been shown to increase (*p* < 0.05) for ewes that received Se-NP, Zn-NP, or SZ-NP vs. control. However, the concentration of milk constituents (%) remained unaffected (*p* > 0.05) among all dietary treatments.

### 3.4. Serum Immunoglobulin G

Serum IgG concentrations in ewes and their offspring at 12, 24, and 48 h post-lambing, as illustrated in Table 5, showed significant enhancement (*p* < 0.05) in all nanoparticle groups compared to the respective controls. Also, ewes that received Se-NP or SZ-NP, and their respective lambs, showed higher (*p* < 0.05) serum IgG concentrations than those that received Zn-NP. Peak serum IgG concentrations occurred at 24 h post-suckling in both ewes (Se-NP: 11.83; Zn-NP: 10.50; SZ-NP: 12.06 mg/mL) and lambs (Se-NP: 11.96; Zn-NP: 10.87; SZ-NP: 12.45 mg/mL), representing 20–27% increases over control groups (ewes: 9.82; lambs: 10.11 mg/mL) followed by a 1.67–8.04% decline by 48 h.

### 3.5. Serum Biochemical Indices

The dietary inclusion of selenium nanoparticles (Se-NP), zinc nanoparticles (Zn-NP), or their combination (SZ-NP) significantly influenced serum biochemical profiles in ewes and their lambs (Table 6). Both Se-NP and SZ-NP enhanced (*p* < 0.05) serum total protein, albumin, and globulin concentrations in ewes and their offspring compared to Zn-NP or control groups, with similar improvements observed for glucose levels. In contrast, cholesterol concentrations showed a non-significant reduction (*p* > 0.05) in Se-NP and SZ-NP groups vs. Zn-NP or control groups. It was noticeably clear that liver health markers (ALT and AST) and urea concentrations were decreased (*p* < 0.05) in all nanoparticle-treated groups vs. the control group. Also, these enzymes and urea show lower (*p* < 0.05) concentrations for ewes that received Se-NP or SZ-NP, and their respective lambs, than those that received Zn-NP.

### 3.6. Thyroid Hormones and Antioxidant Status

Profiles of the serum thyroid hormone concentrations and antioxidant status of ewes and their suckling lambs are illustrated in Table 7. Nanoparticle treatments elevated (*p* < 0.05) serum triiodothyronine (T_3_) and thyroxine (T_4_) concentrations in dams and lambs compared to their respective control. The levels of T_3_ and T_4_ hormones were observed to be higher (*p* < 0.05) for ewes that received Se-NP or SZ-NP, and their lambs, than those of Zn-NP alone. Meanwhile, the values of T_3_/T_4_ ratio did not change among the treatments. In addition, ewes that received Se-NP, Zn-NP, or SZ-NP, and their offspring, have a higher (*p* < 0.05) total antioxidant capacity (TAC) and glutathione peroxidase (GSH-Px) activity than those of the respective controls. It was also observed that Se-NP alone or plus Zn-NP increased the values of TAC and GSH-Px activity (*p* < 0.05) for both ewes and their respective offspring compared to those of Zn-NP alone.

## 4. Discussion

As far as the nutrients’ digestibility and nutritive values are concerned, it was clear that they were significantly improved in ewes that received Se-NP and Zn-NP separately or in combination (SZ-NP). These findings are partially consistent with earlier work in dairy cows [37], which observed that Se improved ADF and NDF digestibility, likely via boosting enzyme activity and rumen microbe population [38]. Recent research confirms this benefit in ewes, showing significantly greater (*p* < 0.05) digestibility of DM, OM, CP, EE, CF, ADF, NDF, and nutritive values (DCP, TDN) with feeding 0.3 mg Nano-Se/kg DM compared to a basal diet [39,40]. Such interpretation demonstrates that moderate Nano-Se dosing (0.3 mg Se/kg DM) effectively improved nutrient digestion [41]. In the same way, Zn-NP supplementation was also reported to significantly increase DM, OM, EE, and NDF digestibility in young Holstein calves fed Zn-NP at 50 mg/kg DM [42]. Similarly, Zn-NP induced a positive effect on DM and OM digestibility in ewes [25,43]. The improvement with feeding Zn-NP could be ascribed to the promotion of Zn’s effect on pancreatic digestive enzymes via protecting the pancreatic tissue against oxidative damage, helping the secretion of pancreatic digestive enzymes, and thereby improving the nutrients’ digestibility [1]. These findings are compatible with [29,44], who found that in vitro DM digestibility was higher with diets containing 20–80 mg Nano-Zn/kg DM than the control, and this dose may be adequate to improve ruminal fermentation [44], as Zn-NP has superior bioavailability, enhancing the population of ruminal microbes, so that nutrients are broken down, leading to improved DM digestibility [45]. Furthermore, zinc could enhance the digestion of complex ruminal substrates by promoting coordinated enzyme action—either individually, synergistically, or via multienzyme complexes [46]—thereby increasing nutrient digestibility. Taken together, the observed improvements in nutrient digestibility, with feeding Se- or Zn- nanoparticles in the presented study, may stem from their nanoscale characteristics such as a high ratio of surface area-to-volume, small size, rapid/specific mobility, antioxidant/anti-inflammatory properties, and catalytic activity, thereby collectively boosting bioavailability through improved absorption in the gastrointestinal tract [24,28].

We found that IBW, FBW, and DMI did not differ among treated ewes (*p* > 0.05); Se-NP and Zn-NP groups showed higher (*p* < 0.05) intakes of DCP and TDN versus controls. These findings are in line with dairy cow studies that observed no effect of Nano-Se or Nano-Zn on DMI [41,47]. Similarly, Nano-Se supplementation did not affect IBW, FBW, or DMI in Awassi or Ossimi ewes [39,48]. Likewise, DMI was unchanged with feeding Nano-Zn in Ossimi ewes [43]. Collectively, the present results may suggest that the inclusion of Se- or Zn-nanoparticles in the ewes’ diet at doses of 0.3 and 30 mg/kg DM, respectively, do not adversely affect palatability and, consequently, the DMI.

In the case of the lambs from the treated ewes, birth weight exhibited an increase by 6.64–43.24% (*p* < 0.05), with Se-NP and SZ-NP groups showing 14.61–20.15% greater BW than Zn-NP alone. Final BW and ADG were increased (*p* < 0.05) by 19.78–35.17% and 21.30–33.52%, respectively, in all supplemented groups, with Se-containing treatments (Se-NP/SZ-NP) outperforming Zn-NP by 8.67–12.85%. The positive results concerning adding Nano-Se are reinforced by other works. For instance, [39] observed significant (*p* < 0.05) enhancements in lamb growth metrics such as BW, FBW, and ADG by 31.84, 25.32, and 23.74%, respectively, when ewes were fed 0.3 mg Nano-Se vs. controls. They discuss that this improvement stems primarily from Se’s critical function in activating the selenoprotein enzyme 5′-deiodinase, to convert T_4_ to the active T_3_ hormone, a process susceptible to Se status, explaining the Se-positive effect for growth and productivity [39,49]. In addition, the observed improvement in ADG could be related to Se’s role in enhancing immunity and its ability to improve energy utilization efficiency for growth. [50,51]. Further contributing factors include the efficient transfer of Se from mother to offspring via the placenta and milk, promoting their immunity and growth [23,50]. These results are compatible with earlier studies [40,52]. The dietary inclusion of Zn-NP increased (*p* < 0.05) ADG of lambs from ewes that received Zn- or SZ-nanoparticles vs. the control. These results are consistent with [42]. They noticed that ADG was shown to be higher in calves that were fed supplementary Zn-nanoparticles vs. control. Also, the potential of Nano-Zn for improving ADG was documented in other studies in ewes or Barki sheep [43,53,54]. Such improvement may be related to the crucial Zn role in several enzymes, hormones, and structural proteins that serve as coenzymes and activators involved in physiological functions, such as growth [15,17]. Furthermore, the increment in ADG observed in the presented study could be ascribed to the beneficial effects of Nano-Zn for enhancing nutrient digestibility [16,24,43].

The mechanism by which supplemental Nano-Se or -Zn positively improved milk production appears to be mediated through multiple mechanisms: improved nutrient digestibility (Table 2), serum metabolites (Table 6), and antioxidant status (Table 7). It is well known that glucose is a lactose precursor and an osmotic component of milk, which raises up the water secretion and subsequently improves milk production [55]. In addition, the inclusion of Se-NP or Zn-NP in the ewes’ diet, as mentioned above, increases their serum metabolic profile, in particular serum albumin and glucose concentrations, which are concomitant with boosting milk production and positive energy balance [56]. Furthermore, Nano-Se is crucial for ewes to enhance their immune function [57], acting as an oxygen-free radical scavenger [58], and boosting intestinal absorption [59], that reflects on optimizing milk production. These results are reinforced by earlier studies showing increased milk, fat, and protein yields in Nano-Se-supplemented dairy cows [37,41] and Ossimi ewes [39] as well as in Nano-Zn-supplemented ewes [43,60]. However, there were no significant changes noticed in milk constituents (g/kg) among treatments. These results are supported by some studies showing that Nano-Se [39,41,47] or Nano-Zn [25,60,61] supplementation did not affect milk constituents of small ruminants.

Ewes receiving Se-NP, Zn-NP, or SZ-NP showed higher IgG levels that were correspondingly reflected in their suckling lambs. This effect was particularly pronounced in the Se-NP and SZ-NP groups, which exhibited superior IgG concentrations relative to Zn-NP alone. This finding indicates that selenium was more potent in promoting passive immunity transfer from the dam to the offspring during critical postnatal hours. Also, adding Se could boost lymphocyte proliferation, potentially accounting for the elevated IgG levels observed in the present study [62]. Similar results were reported with supplemental Nano-Se in suckling lambs [52] or Ossimi ewes and their offspring [39]. In the same way, Nano-Zn has been shown to increase IgG levels in Ossimi ewes and their suckling lambs [43], and in Barki lambs [54] and Ossimi lambs [26]. These findings may be explained by the fact that Zn boosts the replication and proliferation of T and B lymphocytes, leading to the formation of immunoglobulin cells [63,64]. Therefore, Zn is vital for immune cell development and, so, dietary Zn supplementation could enhance immunoglobulin levels [2,18,61].

Serum biochemical profiles serve as sensitive and rapid indicators of nutritional issues and herd health status in ruminant production systems. It is important to mention that the levels of serum biochemical indices in this study were within the normal physiological limits [65], signifying proper nutrition and metabolic function. The significant increase in serum total protein, albumin, and globulin in ewes that received Se-NP or SZ-NP may indicate enhanced utilization of dietary amino acids for protein synthesis. The improved nutrient digestibility supported this, and the increased lamb body weights were observed when dams received Se-NP or SZ-NP (Table 2 and Table 3). Indeed, the dietary inclusion of Nano-Se in the ewes’ diet may boost the efficiency of protein utilization and enhance ruminal fermentation and nutrient digestibility [10,66]. A similar response with supplemental Nano-Se has been shown to positively influence blood metabolite concentration in dairy cows [41]. In the same trend, it has been documented that serum concentrations of total protein, albumin, and globulin are increased (*p* < 0.05) for ewes supplemented with Nano-Se and their respective offspring vs. the control [39]. The results of adding Nano-Zn into the ewes’ diet (Table 6) are consistent with some studies that reported that dietary Nano-Zn supplementation did not affect serum total protein, albumin, and globulin concentrations [67,68]. These results could be discussed as the vital role of Zn in maintaining normal blood protein levels, including albumin and globulin, which are mediators of physiological functions encompassing immunity and osmotic control [69].

Serum glucose levels exhibited an increase (*p* < 0.05) for ewes that received Se-NP or SZ-NP and their suckling lambs vs. Zn-NP or the respective control. The results concerning Nano-Se are reinforced by other works that found higher serum glucose levels with supplemental nano-Se in Holstein dairy cows or calves [41,70]. However, some studies showed that dietary Se supplementation had no effect on the serum glucose of dairy goats or Ossimi ewes [39,71]. The results of serum glucose with the Zn-NP feed were in accordance with those reported in studies of Barki sheep or lactating cows [54,68], in which supplemental nano-Zn did not affect serum glucose concentrations. These findings could be discussed in the way that Zn participates in insulin synthesis, secretion, and signaling, thereby supporting insulin sensitivity for adequate blood glucose regulation; so, sufficient zinc maintains stable blood glucose, preventing hypoglycemia or hyperglycemia that can negatively impact milk production [72].

The decreased levels of total cholesterol for ewes that received Se-NP and SZ-NP and their suckling lambs vs. Zn-NP or the respective control may signify a beneficial effect of the dietary inclusion of Se-NP in the ewes’ diet upon energy metabolism and lipid regulation [73]. Such lowered cholesterol levels may also reflect increased energy demands linked to the animals’ physiological status [74]. This interpretation is consistent with some studies working on sheep [39,75], where nano-Se was able to decrease serum cholesterol levels. The lack of the inclusion of the Zn-NP effect on serum cholesterol levels was consistent with a study that found that adding 25 or 50 mg nano-Zn/kg DM to growing heifers had no significant effect on their serum cholesterol levels [63]. A similar trend of blood cholesterol level was also noticed in lactating cows fed 20 mg nano-Zn [68].

It was noticeably clear that serum concentrations of ALT and AST were decreased (*p* < 0.05) for both ewes that received Se-NP or SZ-NP and their suckling lambs vs. Zn-NP alone. These findings could be mediated by the Se role in eliminating hydrogen peroxide through enhancing glutathione peroxidase activity, potentially reducing elevated levels of liver damage markers, including AST and ALT. As well, Se-NP exhibits anti-inflammatory properties, which may help alleviate hepatic inflammation and improve overall liver function [76]. This result was also noticed by some studies showing a decrease in AST and ALT activities with Nano-Se supplementation in sheep [39,74,75,77]. However, in the case of inclusion of Zn-NP in the ewes’ diet, it did not affect ALT and AST activities (Table 6). The lack of Zn-NP effect on ALT and AST levels was in good agreement with studies working on sheep [25,43,53], cattle [63], and lactating cows [68], where the values of these enzymes were within the normal physiological ranges [78]. Taken together, this may indicate that the experimental diets used in this study had no adverse effects, or any hepatocellular damage, due to the dietary inclusion of Se-NP or Zn-NP. Moreover, Se and Zn may promote hepatic health by enabling efficient protein metabolism and helping prevent liver disorders [68]. It is well known that the rise in the activities of such enzymes can be an indication of elevated liver function due to hepatic injury [79].

The finding of lower serum urea concentrations observed in ewes that received Se-, Zn-, or, SZ-NP and their offspring vs. the respective control may indicate enhanced renal function. Nano-Se may assist enhanced renal function by lowering oxidative stress and inflammation in the kidneys, enhancing antioxidant status, mitigating the harmful effects of urea, and minimizing kidney damage [80]. The current results are supported by similar observations in Ossimi ewes and dairy cows supplemented with Nano-Se [39,41,81]. Ewes received 30 mg Zn-NP and their offspring had lower serum urea levels than the respective control (Table 6). In the same way, supplemental Nano-Zn (28 mg/kg DM) has been shown to decrease blood urea level in sheep [82]. A similar trend of reduced serum urea levels was noticed in Barki lambs supplemented with 15 and 30 mg Zn-NP [53]. On the other hand, no significant changes in blood urea concentrations in heifers receiving 25–50 mg or lactating cows supplemented with 20 mg nano-Zn [63,68].

Serum concentrations of T_3_ and T_4_ hormones were increased (*p* < 0.05) for both ewes that received Se-NP, Zn-NP, or SZ-NP and their lambs vs. the respective control. These results were reinforced by a study that observed that supplemented pregnant ewes with Nano-Se at 0.1 and 0.2 mg/kg DM significantly elevated T_3_ and T_4_ levels in their suckling lambs by 36.25 and 45.31, and 2.08 and 9.4%, respectively, vs. the control group [52]. A comparable increase in T_3_ and T_4_ levels was noticed in Ossimi ewes fed 0.3 mg Nano-Se/kg DM [39]. The Se role could mediate the improvements in T_3_ and T_4_ hormones as a structural component of selenocysteine, a critical moiety in thioredoxin reductase and iodothyronine deiodinase enzymes, both of which are essential for thyroid hormone metabolism, and catalyze activation of T_3_ from T_4_ [7,8,49]. In the case of adding Zn-NP, it had enhanced T_3_ and T_4_ hormones in ewes and their offspring group vs. the respective control (Table 6). This finding may be explained by the fact that Zn plays catalytic, structural, and regulatory roles in enzymes, proteins, and transcription factors [14]. As well, Zn is involved in metabolism and growth [15,16], with approximately 300 enzymes that are related to metabolism and other biochemical processes [17]. Similar results were documented following Zn supplementation in goat bucks and Ossimi ewes [43,83]. However, some studies showed no effect of supplemental nano-Zn on T_3_ and T_4_ profiles in goats [84].

Serum TAC and GSH-Px activity were higher for ewes that received Se-NP and Zn-NP separately or in combination (SZ-NP), and their suckling lambs vs. the respective control. It has been reviewed that the mechanism, by which nanominerals, especially Se and Zn, enhance antioxidant indicators, could be ascribed as nanominerals and may promote antioxidant activity via inhibiting the free radicals due to the increased surface area, leading to a higher number of active sites for scavenging an increased number of free radicals [24]. These findings are in line with the previous studies, which indicated that supplemental Se increased TAC concentrations and GSH-Px activity in small ruminants [3,40]. In respect to Zn-NP, the results were further reinforced by the study conducted by [42], who reported that TAC activity was greater in calves supplemented with 50 mg Nano-Zn. Similarly, TAC levels exhibited higher values with supplemental Nano-Zn in Barki lambs and Ossimi lambs [26,53]. This increment in TAC may be attributed to the ability of Nano-Zn, as an antioxidant, which helps the body rid itself of free radicals [25], or indirectly prevents free radical formation and slows oxidative processes. Since Zn plays a crucial role in the formation of metallothionein and acts as a free radical scavenger [85].

Furthermore, the biosafety of the dietary inclusion of Se-NP and Zn-NP in the ewes’ diet is supported by the absence of adverse effects on key health biomarkers. The doses of Se-NP (0.3 mg/kg diet) and Zn-NP (30 mg/kg diet) were determined to be safe for inclusion in sheep diets based on the results of the present study. This conclusion is evidenced by the stability of liver enzyme markers (AST and ALT), indicating no hepatotoxicity, and unchanged urea concentration, suggesting no compromised renal function. Moreover, the significant improvement in antioxidant status, as indicated by enhanced GSH-Px activity and TAC, confirms that the nanoparticles provided a beneficial physiological effect without inducing oxidative stress, which is a common concern with higher metal nanoparticle doses. Collectively, our results provide strong evidence for the livestock industry to replace inorganic minerals with a combination of nano-selenium and nano-zinc, offering a practically feasible way to enhance ewe metabolism and lamb performance within existing feeding systems. While this study demonstrates these positive effects on productivity and health indices, it is important to acknowledge its limitations; specifically, serum selenium and zinc concentrations were not measured, and this does not allow us to know whether a deficiency is being corrected, as our primary objective was to assess downstream functional outcomes rather than mineral absorption kinetics. Nevertheless, the significant improvements observed provide strong indirect evidence of the bioavailability and efficacy of the nanoparticle supplements. For farmers, this translates to a viable solution for improving flock productivity and health. To build upon these findings, future research should focus on determining the optimal dosage across different sheep breeds and production systems, and it must prioritize the direct quantification of absorption and bioavailability through the analysis of serum, tissue, and fecal mineral levels to precisely correlate these with functional improvements. Ultimately, investigating the long-term environmental impact of nano-mineral excretion will be crucial to ensuring the sustainability of this approach.

However, the broader applicability of these results should be interpreted considering the study’s limitations, including its restriction to a single breed (Ossimi) and the need for validation in larger, more diverse flocks under various management conditions. Future research should, therefore, focus on validating these outcomes in larger-scale trials and across different sheep breeds; conduct a thorough economic analysis to determine the cost–benefit ratio of adopting nano-mineral supplementation; and investigate the molecular mechanisms underlying the observed synergistic effects between selenium and zinc nanoparticles on nutrient metabolism and immune function.

## 5. Conclusions

Based on the present results, it can be concluded that dietary supplementation of Ossimi ewes, at the late gestation and suckling period, with 0.3 mg Nano-Se, 30 mg Nano-Zn, or their combination, can positively improve nutrient digestibility, productive performance, serum biochemical indices, and antioxidant status. This improvement was accompanied by better growth performance, immune function, and overall health in their suckling lambs. These findings demonstrate the practical applicability of nano-mineral supplementation, particularly the combined Nano-Se and Nano-Zn treatment, as a viable strategy to enhance productivity and health in sheep production systems.

## Figures and Tables

**Table 1:** Proximate analysis of concentrate feed mixture, alfalfa hay, and total mixed ration fed to Ossimi ewes (% on DM basis).

Items	CFM	AH	TMR
DM	88.92	84.20	87.36
OM	92.24	88.51	91.01
CP	17.88	14.15	16.65
EE	3.45	1.04	2.66
CF	10.58	24.98	15.33
NFE	60.32	48.33	56.37
NDF	26.26	60.20	37.45
ADF	13.89	48.34	25.24
Ash	7.76	11.49	8.99

CFM: Concentrate feed mixture; AH: Alfalfa hay; TMR: Total mixed ration.

**Table 2:** Effects of the dietary inclusion of Se, Zn nanoparticles, or their combination on nutrient digestibility and nutritive values of experimental rations.

Items	Treatments					
	Control	Se-NP	Zn-NP	SZ-NP	SEM	*p*-Value
Nutrients digestibility (%)						
DM	71.35 ^c^	78.44 ^a^	74.29 ^b^	80.62 ^a^	0.38	0.006
OM	72.87 ^c^	79.21 ^a^	7596 ^b^	81.50 ^a^	0.37	0.008
CP	71.20 ^c^	78.27 ^a^	74.52 ^b^	78.65 ^a^	0.35	<0.0001
EE	69.83 ^c^	78.82 ^a^	73.45 ^b^	79.09 ^a^	0.16	<0.0001
CF	64.09 ^b^	67.64 ^a^	64.99 ^b^	68.69 ^a^	0.43	0.002
NDF	53.14 ^b^	56.39 ^a^	53.63 ^b^	57.81 ^a^	0.33	0.0003
ADF	43.75 ^b^	46.94 ^a^	44.35 ^b^	47.41 ^a^	0.66	0.039
Nutritive values (%)						
DCP	11.86 ^c^	13.03 ^a^	12.41 ^a^	13.10 ^a^	0.06	<0.0001
TDN	64.72 ^c^	68.45 ^a^	66.61 ^a^	68.96 ^a^	0.37	0.013

^a, b, c^ Values within a row with different letters differ significantly (*p* < 0.05); Se-NP = Selenium nanoparticles (0.3 mg/kg DM); Zn-NP = Zinc nanoparticles (30 mg/kg DM); SZ-NP = Se-NP plus Zn-NP.

**Table 3:** Effects of the dietary inclusion of Se, Zn nanoparticles, or their combination on growth performance of ewes and their suckling lambs.

Items	Treatments					
	Control	Se-NP	Zn-NP	SZ-NP	SEM	*p*-Value
Ewes:						
IBW (late-gestation), kg	49.56	49.25	50.02	49.88	1.62	0.354
FBW (kg/day)	46.17	47.80	47.96	48.33	1.04	0.368
AHI (kg/day)	0.58	0.61	0.62	0.61	0.02	0.346
CFMI (kg/day)	1.18	1.18	1.19	1.20	0.04	0.387
DMI (kg/day)	1.76	1.79	1.81	1.81	0.05	0.288
DCPI (g/head/day)	208.74 ^c^	233.24 ^a^	224.62 ^b^	237.11 ^a^	0.87	˂0.0001
TDNI (g/head/day)	1139.07 ^c^	1225.26 ^a^	1205.64 ^b^	1248.18 ^a^	5.48	0.012
Lambs:						
Birth weight (BW, kg)	3.33 ^c^	4.55 ^a^	3.97 ^b^	4.77 ^a^	0.11	0.0005
FBW (kg)	18.00 ^c^	23.86 ^a^	21.56 ^b^	24.33 ^a^	0.16	0.0009
ADG (g/day)	195.33 ^c^	257.47 ^a^	236.93 ^b^	260.80 ^a^	4.34	0.003

^a, b, c^ Values within a row with different letters differ significantly (*p* < 0.05); Se-NP = Selenium nanoparticles (0.3 mg/kg DM); Zn-NP = Zinc nanoparticles (30 mg/kg DM); SZ-NP = Se-NP plus Zn-NP.

**Table 4:** Effects of the dietary inclusion of Se, Zn nanoparticles, or their combination on milk production and its composition in Ossimi ewes (Mean ± SEM).

Items	Treatments					
	Control	Se-NP	Zn-NP	SZ-NP	SEM	*p*-Value
Milk yield (g/head/day)	376.84 ^c^	556.02 ^a^	499.30 ^b^	562.14 ^a^	0.04	˂0.0001
FCM (g/head/day)	391.48 ^c^	586.07 ^a^	522.49 ^b^	594.66 ^a^	0.05	˂0.0001
DMI (kg/day)	1.76	1.79	1.81	1.81	0.87	0.288
FCM/TDMI	0.22 ^c^	0.33 ^a^	0.29 ^b^	0.33 ^a^	5.48	0.007
Milk composition (%)						
Fat	6.43	6.59	6.51	6.63	0.08	0.621
Protein	4.87	5.08	4.95	5.13	0.15	0.851
Lactose	4.64	4.97	4.68	5.01	0.12	0.910
Solid not fat	10.28	10.84	10.41	10.95	0.21	0.233
Ash	0.77	0.79	0.78	0.81	0.02	0.705
Total solids	16.71	17.43	16.92	17.58	0.17	0.425
Milk constituents yield (g/d)						
Fat	24.23 ^c^	36.64 ^a^	29.25 ^b^	37.27 ^a^	0.20	˂0.0001
Protein	18.35 ^c^	28.25 ^a^	22.24 ^b^	28.84 ^a^	0.23	˂0.0001
Lactose	17.49 ^c^	27.63 ^a^	21.03 ^b^	28.16 ^a^	0.11	˂0.0001

^a, b, c^ Values within a row with different letters differ significantly (*p* < 0.05); Se-NP = Selenium nanoparticles (0.3 mg/kg DM); Zn-NP = Zinc nanoparticles (30 mg/kg DM); SZ-NP = Se-NP plus Zn-NP.

**Table 5:** Effects of the dietary inclusion of Se, Zn nanoparticles, or their combination on serum immunoglobulin G (IgG, mg/mL) concentrations of Ossimi ewes and their suckling lambs.

Items	Treatments					
	Control	Se-NP	Zn-NP	SZ-NP	SEM	*p*-Value
Ewes						
12 h	7.99 ^c^	9.81 ^a^	8.35 ^b^	9.97 ^a^	0.07	˂0.0001
24 h	9.82 ^c^	11.83 ^a^	10.50 ^b^	12.06 ^a^	0.10	˂0.0001
48 h	9.03 ^c^	11.54 ^a^	9.96 ^b^	11.80 ^a^	0.11	˂0.0001
Lambs						
12 h	8.43 ^c^	10.17 ^a^	9.23 ^b^	10.63 ^a^	0.11	˂0.0001
24 h	10.11 ^c^	11.96 ^a^	10.87 ^b^	12.45 ^a^	0.04	˂0.0001
48 h	9.57 ^c^	11.76 ^a^	10.55 ^b^	11.91 ^a^	0.08	˂0.0001

^a, b, c^ Values within a row with different letters differ significantly (*p* < 0.05); Se-NP = Selenium nanoparticles (0.3 mg/kg DM); Zn-NP = Zinc nanoparticles (30 mg/kg DM); SZ-NP = Se-NP plus Zn-NP.

**Table 6:** Effects of the dietary inclusion of Se, Zn nanoparticles, or their combination on some serum biochemical indices of Ossimi ewes and their suckling lambs.

Items	Treatments					
	Control	Se-NP	Zn-NP	SZ-NP	SEM	*p*-Value
Ewes						
Total protein (g/dL)	6.85 ^b^	8.35 ^a^	7.33 ^b^	8.52 ^a^	0.31	0.023
Albumin (g/dL)	3.69 ^b^	4.32 ^a^	3.94 ^b^	4.57 ^a^	0.22	0.014
Globulin (g/dL)	3.16 ^b^	4.03 ^a^	3.39 ^b^	3.95 ^a^	0.27	0.05
A/G ratio	1.18	1.07	1.16	1.16	0.13	0.342
Glucose (mg/dL)	82.18 ^b^	95.47 ^a^	82.54 ^b^	94.08 ^a^	1.45	0.003
Cholesterol (mg/dL)	105.42	98.37	101.41	97.83	2.43	0.133
AST (U/L)	54.00 ^a^	33.08 ^c^	49.20 ^b^	31.58 ^c^	2.53	0.002
ALT (U/L)	25.67 ^a^	19.00 ^c^	23.86 ^b^	20.50 ^c^	0.97	0.001
Urea (mg/dL)	70.78 ^a^	40.14 ^c^	54.15 ^b^	38.89 ^c^	2.17	0.009
Suckling Lambs						
Total protein (g/dL)	6.65 ^b^	7.57 ^a^	7.10 ^b^	7.74 ^a^	0.09	0.0003
Albumin (g/dL)	3.38 ^b^	3.87 ^a^	3.54 ^b^	3.94 ^a^	0.05	0.0004
Globulin (g/dL)	3.27 ^b^	3.69 ^a^	3.56 ^b^	3.80 ^a^	0.11	0.029
A/G ratio	1.04	1.05	0.99	1.04	0.04	0.956
Glucose (mg/dL)	83.77 ^b^	90.72 ^a^	86.09 ^b^	92.90 ^a^	1.57	0.014
Cholesterol (mg/dL)	105.17	98.16	102.69	97.19	2.19	0.017
AST (U/L)	44.67 ^a^	38.02 ^c^	42.25 ^b^	35.75 ^c^	0.45	˂0.0001
ALT (U/L)	25.58 ^a^	20.42 ^c^	23.00 ^b^	19.58 ^c^	1.87	0.013
Urea (mg/dL)	64.41 ^a^	33.43 ^c^	48.17 ^b^	32.50 ^c^	0.93	˂0.0001

^a, b, c^ Values within a row with different letters differ significantly (*p* < 0.05); Se-NP = Selenium nanoparticles (0.3 mg/kg DM); Zn-NP = Zinc nanoparticles (30 mg/kg DM); SZ-NP = Se-NP plus Zn-NP.

**Table 7:** Effects of the dietary inclusion of Se, Zn nanoparticles, or their combination on serum thyroid hormone concentrations and antioxidant status of Ossimi ewes and their suckling lambs.

Items	Treatments					
	Control	Se-NP	Zn-NP	SZ-NP	SEM	*p*-Value
Ewes						
T_3_ (nmol/L)	2.23 ^c^	2.90 ^a^	2.74 ^b^	2.97 ^a^	0.04	0.017
T_4_ (nmol/L)	41.63 ^c^	62.30 ^a^	53.06 ^b^	64.49 ^a^	1.27	0.003
T_3_/T_4_	0.054	0.047	0.052	0.046	0.79	0.476
TAC (mM/L)	1.86 ^c^	3.13 ^a^	2.45 ^b^	3.37 ^a^	0.06	0.038
GSH-Px (U/mL)	87.87 ^c^	153.35 ^b^	128.01 ^b^	165.29 ^a^	3.26	˂0.0001
Suckling Lambs						
T_3_ (nmol/L)	2.55 ^c^	3.74 ^a^	3.28 ^b^	3.82 ^a^	0.06	˂0.0001
T_4_ (nmol/L)	46.96 ^c^	76.07 ^a^	65.17 ^b^	77.62 ^a^	1.98	0.005
T_3_/T_4_	0.050	0.049	0.054	0.049	1.41	0.541
TAC (mM/L)	2.38 ^c^	3.81 ^a^	3.29 ^b^	3.94 ^a^	0.07	0.001
GSH-Px (U/mL)	82.13 ^c^	156.70 ^a^	107.06 ^b^	154.55 ^a^	3.18	˂0.0001

^a, b, c^ Values within a row with different letters differ significantly (*p* < 0.05); Se-NP = Selenium nanoparticles (0.3 mg/kg DM); Zn-NP = Zinc nanoparticles (30 mg/kg DM); SZ-NP = Se-NP plus Zn-NP. TAC = total antioxidant capacity; GSH-Px = glutathione peroxidase.

## Data Availability

The data that support the findings of this study are available on request from the corresponding authors.
